# Inferring domain-domain interactions from protein-protein interactions in the complex network conformation

**DOI:** 10.1186/1752-0509-6-S1-S7

**Published:** 2012-07-16

**Authors:** Chen Chen, Jun-Fei Zhao, Qiang Huang, Rui-Sheng Wang, Xiang-Sun Zhang

**Affiliations:** 1 Academy of Mathematics and Systems Science, Chinese Academy of Sciences, Beijing 100190, PR China; 2 Department of Physics, Pennsylvania State University, University Park, PA 16802, USA; 3 National Center for Mathematics and Interdisciplinary Sciences, Chinese Academy of Sciences, Beijing 100190, PR China

## Abstract

**Background:**

As protein domains are functional and structural units of proteins, a large proportion of protein-protein interactions (PPIs) are achieved by domain-domain interactions (DDIs), many computational efforts have been made to identify DDIs from experimental PPIs since high throughput technologies have produced a large number of PPIs for different species. These methods can be separated into two categories: deterministic and probabilistic. In deterministic methods, parsimony assumption has been utilized. Parsimony principle has been widely used in computational biology as the evolution of the nature is considered as a continuous optimization process. In the context of identifying DDIs, parsimony methods try to find a minimal set of DDIs that can explain the observed PPIs. This category of methods are promising since they can be formulated and solved easily. Besides, researches have shown that they can detect specific DDIs, which is often hard for many probabilistic methods. We notice that existing methods just view PPI networks as simply assembled by single interactions, but there is now ample evidence that PPI networks should be considered in a global (systematic) point of view for it exhibits general properties of complex networks, such as 'scale-free' and 'small-world'.

**Results:**

In this work, we integrate this global point of view into the parsimony-based model. Particularly, prior knowledge is extracted from these global properties by plausible reasoning and then taken as input. We investigate the role of the added information extensively through numerical experiments. Results show that the proposed method has improved performance, which confirms the biological meanings of the extracted prior knowledge.

**Conclusions:**

This work provides us some clues for using these properties of complex networks in computational models and to some extent reveals the biological meanings underlying these general network properties.

## Background

Recently, researchers have confirmed that most proteins perform their functions through physically binding to other proteins, permanently or transiently. These interactions can be represented as a protein-protein interaction (PPI) network with each node corresponding to a protein and each edge an interaction. The development of high-throughput technologies, such as yeast two-hybrid screening methods [1,2] and affinity purification with mass spectroscopy [3,4], has produced numerous data of protein-protein interactions for different species, which provides us an opportunity to investigate cellular processes in a systematic view.

In general, proteins consist of one or more structural domains. A PPI is usually carried out through domain-domain interactions (DDIs). While PPIs are not so conserved among species, the recognition patterns of domain pairs are often shared within organisms [5]. Knowledge about domain-domain recognition patterns can provide us a deeper understanding of the interaction network of proteins. Since interactions between domains are difficult to be determined experimentally, many computational approaches have been proposed to discover DDI patterns from experimental PPIs.

From a computational perspective, these methods fall into two categories. In the first category, they try to find pairs of domains that co-occur significantly more often in interacting protein pairs than in non-interacting pairs. The association method [6] computes a score for every domain pair according to the ratio of its occurrences in interacting protein pairs to non-interacting pairs. Deng and colleagues [7] extended this idea to a more sophisticated probabilistic model in which they applied an expectation maximization algorithm to predict interacting domains consistent with observed PPIs. Riley and colleagues [8] found that previous probabilistic models cannot detect specific interactions very well. A specific DDI means that domain *i *and domain *j *may interact in a context-depended way, so observed interactions and non-interactions including *i *and *j *are not always exclusive. In order to detect specific interactions, they introduced an E-value, which measures to what extent a given domain pair cannot be replaced by another pair.

The second category, different from the probabilistic framework, often models the issue as a combinatorial optimization problem. The idea is that an observed PPI can be explained by at least one pair of interacting domains involved, then they try to explain observed interacting protein pairs using a minimal number of domain pairs (the minimal spanning set), namely, the parsimony based approaches [9-11]. These methods do not treat unobserved PPIs as evidence of non-interaction of domain pairs involved, and therefore specific interactions can be detected easily. Furthermore, parsimony-based models can be formulated as an integer linear programming and then relaxed to a linear programming problem, which has efficient algorithms to solve.

Although the problem is thoroughly studied these years, we realize that existing models only make use of the local information of PPI networks (assembled single interactions). There is now ample evidence that PPI networks should be considered in a global (systematic) point of view for it exhibits some general properties of complex networks. 'Complex Networks' is an emerging concept that unifies networks appearing in different disciplines, such as social networks, information networks, and biological networks [12]. Though these networks are irrelevant at first sight, empirical studies have shown that they share some common properties, such as 'small-world', 'scale-free' and relatively larger clustering coefficient. A 'small-world' network is a network with short characteristic path lengths, like random networks, but still being highly clustered, like regular lattice networks [13]. A 'scale-free' network is a network with power-law degree distribution [14]. The clustering coefficient measures the density of triangles in a network, and it tends to be a non-zero constant when the size of the network grows [12]. Besides, there are some more detailed hidden features of complex networks which have been revealed recently, such as rich-club structure and mixing patterns (assortative mixing or disassortative mixing) [15]. In a network, nodes with large numbers of links are called rich nodes. It is found that rich nodes are connected to each other as a close community, called as rich club, in many social and computer networks. But in PPI networks, rich nodes are loosely connected, i.e., there is no rich club phenomenon [16,17]. Oppositely, rich nodes in PPI networks tend to connect nodes with small degree, a structure called disassortative mixing by node degree. With these clues, we extract prior information by plausible reasoning and integrate them into a parsimony-based model [9]. The modified model shows improved accuracy and we validate the performance difference carefully to confirm that it is a consequence of integrated prior information. This provides us some clues for using these global and common properties of complex networks in computational models and to some extent reveals the biological meanings underlying these network properties.

Besides, although the parsimony principle is widely used in computational biology, few work has been done to verify its rationality quantitatively. Here, we investigate the parsimony nature of the organization of DDIs in mediating PPIs through randomization-based testings, which justifies the parsimony assumption from a computational perspective.

## Methods

### Parsimony based methods

Zhang et al. [9] developed a protein interaction prediction method based on the parsimony principle. In the first step of the method, an integer linear programming model is used to infer domain-domain interactions from given protein interaction data. Guimarães et al. used a parsimony explanation (PE) approach to predict domain-domain interactions from protein interactions [10], in which the model is exactly the same as the basic parsimony model in [9], although two models were carried out independently and implemented differently. We describe the details of the models here.

We denote the observed protein-protein interaction network as *I *= (*P, E*), where *P *= {*P*_1_*, P*_2_,..., *P_N_*} is the set of proteins in the network and *E *is the set of PPIs. *D *= {(*D_i_*, *D_j_*)| *D_i_*∊ *P_m_*, *D_j_*∊ *P_n_*, (*P_m_*, *P_n_*) ∊ *E*} is the set of all possible domain pairs. Zhang et al. gave a formulation as follows to determine a parsimonious core of DDIs:

(1) $$Min:\underset{\left(i,j\right)\in D}{\sum }dij$$

(2) $$st:\underset{\left(i,j\right)\in \left(P_{m},P_{n}\right)}{\sum }d_{ij}+e_{mn}\geq 1,\;\left(P_{m},P_{n}\right)\in E$$

(3) $$\underset{\left(P_{m},P_{n}\right)\in E}{\sum }e_{mn}\leq \left(1-sd\right)\left|E\right|$$

(4) $$d_{ij},e_{mn}\in \left\{0,1\right\}$$

Here, we use (*i*, *j*) ∊ (*P_m_*, *P_n_*) to represent domain pairs involved in the corresponding protein-protein interaction. This is a flexible version of parsimony assumption. The objective function guarantees that as few as domain pairs should be used. The following constraints enables every observed PPI must be explained by at least one involved DDI or by a virtual variable *e_mn_*. When *e_mn_*is set to 1, it is equivalent to deleting the corresponding PPI (*P_m_*, *P_n_*) from the constraints. Then a tuning parameter *sd *is employed to control the proportion of protein interactions that must be explained by DDIs. This model is named as ILP (Integer Linear Programming) model for later quotation.

Guimaraes et al. proposed a model with the same idea as [9], but there is some difference in implementing:

(5) $$Min:\underset{\left(i,j\right)\in D}{\sum }d_{ij}$$

(6) $$st:\underset{\left(i,j\right)\in \left(P_{m}P_{n}\right)}{\sum }d_{ij}\geq 1,\left(P_{m}P_{n}\right)\in E$$

(7) $$d_{ij}\in \left\{0,1\right\}$$

They modeled the noise in the protein-protein interaction data by selecting the constraints randomly according to a reliability probability *r*. For each reliability level, the procedure was performed 1000 times, then the values obtained were averaged to generate the reported LP-score [10]. Besides the LP-score, they introduced a statistical measure for each domain pair, specifically *pw-score*(*i, j*) = *min*{*p-value*(*i, j*), (1 - *r*)^*w*(*i, j*)^}. *P*-value is a measure for evaluating the significance of the LP-score of *d_ij_*, which is computed through a randomization experiment with a set of 1000 random networks as reference. *w*(*i, j*) denotes the number of witnesses (interacting pairs of single-domain proteins supporting it) for *d_ij_*. (1 - *r*)^*w*(*i, j*) ^denotes the probability that all PPIs corresponding to witnesses are false positives. This term is useful for removing promiscuous domain-domain interactions that are scored high only because of their appearance frequency.

The aforementioned methods utilize a common computational assumption, namely, parsimony principle. In fact, the parsimony principle has been widely used in computational biology due to its biological/evolutional implication and intuitive simplicity. For example, parsimony strategy has been used in haplotype inference [18,19] and in phylogenetic tree construction [20] as one of the main modeling methodologies. While the intuition behind the parsimony principle is clear enough, few work has been done to show to what extent the biology data are organized in a parsimonious way. In this paper, we will verify it in the context of predicting DDIs through a computational approach.

### The parsimony essential of PPIs

**Figure 1:**
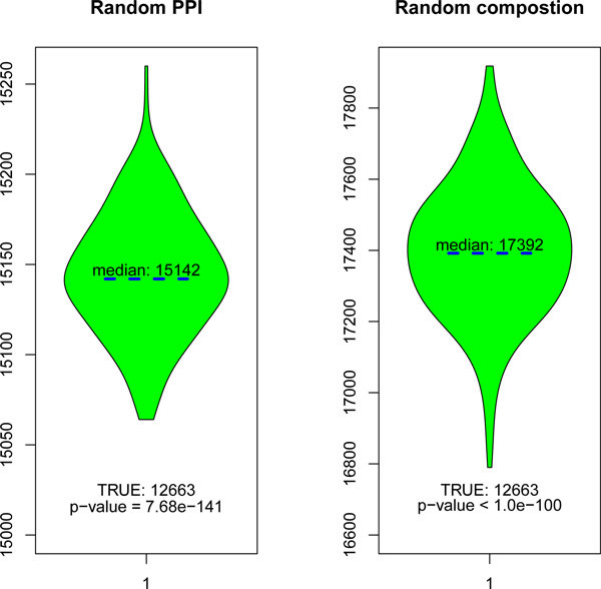
**PPIs and protein domain compositions are parsimoniously organized in nature**. Under each null model, 200 data sets are simulated. The distribution of *T *is shown as 'violin plot' and *p*-value is computed based on Gaussian distribution.

To verify the parsimony assumption in the context of predicting DDIs, we design two randomization testings. The parsimony principle here is to use a minimal number of DDIs to explain the observed PPIs. We define a null model in which there is no evolutional optimization process in organizing the protein domain composition and protein-protein interactions and compute the minimal number of such DDIs through (Eq. 5-7). To achieve this, the original data set is shuffled randomly. In order to simplify the argument, we define a random variable *T *denoting the minimal number of DDIs computed from the shuffled data set, and *T*_0 _is the corresponding value computed from the original data. So, under the null model, we expect to see a significant larger *T *compared with *T*_0_. Particularly, the original data set is shuffled with two different rules. The first rule shuffles the protein domain composition while the PPIs are conserved (For each protein, the number of constituent domains is conserved), and conversely, the second rule shuffles the PPIs while maintaining the composition (the degree distribution of the PPI network is conserved). The PPIs of *Saccharomyces cerevisiae *are employed here (described in detail below), and we have *T*_0 _= 12663 on this data set. The distribution of *T *is shown as 'violin plots' (Figure 1), *p*-values are computed using the Gaussian distribution. There is a significant difference between *T *(under null model) and *T*_0 _(In both cases, *p*-values are smaller than 1.00*e*-100), which confirms the parsimony principle in the context of predicting DDIs. In the following, we modify the model proposed in [9] to integrate the global information of PPI networks, and investigate the performance changes carefully to extract its role.

### Motivation

Considering that it is intractable to directly integrate 'small-world' or 'scale-free' properties into the model as they are both statistical descriptions, we turn to consider the clustering coefficient *C*. Empirical studies have shown that many complex networks possess relatively large clustering coefficient, which we will use as prior information. We describe the definition of *C *proposed by Watts and Strogatz [13] here. For each vertex, a local value of the clustering coefficient is defined as follows:

(8) $$\begin{array}{c} C_{i}=\frac{\text{number}\;\text{of}\;\text{trianngles}\;\text{connected}\;\text{to}\;\text{vertex}\;i}{\text{number}\;\text{of}\;\text{triples}\;\text{centered}\;\text{on}\;\text{vertex}\;i} \end{array}$$

For vertices with degree 0 or 1, both the numerator and denominator are zero, so define *C_i_*= 0. Then the clustering coefficient for the whole network is the average:

(9) $$\begin{array}{c} C=\frac{1}{n}\underset{i}{\sum }C_{i} \end{array}$$

In terms of social networks, a large clustering coefficient implies the friend of your friend is likely also to be your friend. In many real complex networks, the clustering coefficient tends to be a non-zero number when the size of the network grows, while in random networks, it tends to be zero.

In the definition above, nodes with small degree contribute larger values to the global clustering coefficient because they own smaller denominators (Eq. 8), so we can deduce that the existence of triangle structures connected to poor nodes (nodes with few neighbors) plays a crucial role in maintaining relatively large *C*. We can express the idea in another way: if we are allowed to add finite edges into an existing network, in order to maintain or increase the clustering coefficient, it is better to connect nodes adjacent to a same poor node. In the context of protein-protein interaction networks, it means that proteins which share a common neighbor with small degree are expected to be interacting.

We can also think it in a biological way. It is known that most proteins carry out their functions through physically binding to other proteins, rather than in an individual way. So proteins with few neighbors are more likely to form a tight complex with its neighbors, that is to say, its neighbors interact with each other. On the other hand, rich nodes are more likely to execute multiple functions under different cell types/conditions, and experimentally detected interactions associated to rich nodes are the union of these cell type/condition specific interactions, we can not deduce any interaction potential of those proteins connected to a rich node.

Among experimental PPIs, a large proportion are false positives, which hinders many computational models. As discussed above, from a network view and biological intuition, we reason that detected interactions centering on a poor node are more likely to be true positives.

### Weighted integer linear programming model

Based on the discussion above, we give preferences to observed PPIs. Interactions between proteins sharing a poor neighbor have priorities of being explained by DDIs. For such interactions, smaller weights are given to domain pairs involved. The mathematical description is as follows: Suppose *d_min_*(*d_max_*) is the minimum (maximum) degree of the nodes in the protein-protein interaction network. The interval [*d_min_*, *d_max_*] is divided into *K *subintervals *I_k_*(*k *= 1,..., *K*) and every node falls into one subinterval. *I*_1 _contains proteins with small degree while *I_K_*contains most of the hubs. Then for a protein contained in *I*_1 _and an interaction centering on the protein, smaller weights are given to domain pairs involved in the interaction. We define a set of domain pairs as follows: *S *= {*d_ij_|d_ij_*∊ (*P_m_*, *P_n_*), *P_m_*, *P_n_*∊ *N_P_*, *P *∊ *I*_1_, *P_m_*∊ *I_s_, P_n_*∊ *I_t_*}, where *N_P_*contains all the neighbors of protein *P *in the PPI network.

(10) $$w_{ij}=\left\{\begin{array}{cc} \frac{1}{1+\left|s-t\right|} & \text{If}\;d_{ij}\in S;\\ 1 & \text{Otherwise}. \end{array}\right)$$

If *d_ij_*spans more than one interaction (*P_m_, P_n_*), then *w_ij_*takes the smallest value. A larger |*s *- *t*| in the denominator generates a smaller weight, which promote the priority of the corresponding domain pair, consistent with that rich nodes in the PPI network tend to connect nodes with small degree (disassortative mixing).

Then, we get a weighted integer linear programming model (WILP):

(11) $$Min:\underset{\left\{i,j\right\}\in D}{\sum }w_{ij}d_{ij}$$

(12) $$st:\underset{\left(i,j\right)\in \left(P_{m},P_{n}\right)}{\sum }d_{ij}+e_{mn}\geq 1,\left(P_{m},P_{n}\right)\in E$$

(13) $$\underset{\left(P_{m},P_{n}\right)\in E}{\sum }e_{mn}\leq \left(1-sd\right)\left|E\right|$$

(14) $$d_{ij},e_{mn}\in \left\{0,1\right\}$$

This model is named as WILP (Weighted Integer Linear Programming) model for later quotation. In practical computation, the linear integer programming is relaxed to a linear programming by allowing *d_ij_*, *e_mn_*to take continuous values between 0 and 1. It is interesting to notice that when we solve the problem using simplex method, the optimal solutions are almost always with integer components.

## Results and discussion

### Data sets

PPIs of *S.cerevisiae *are downloaded from the DIP database (*Scere20101010*) [21], in which there are 25180 interactions underlying 5173 proteins. The protein domain compositions are extracted from the Pfam database (*Pfam 25.0*) [22], where 4125 of DIP proteins are defined with Pfam-A domains. Finally there are 20709 PPIs that both proteins are defined in the Pfam database. To evaluate the performance of the model, DDIs in the *iPfam *[23] and *3did *[24] databases are collected to form a golden standard data set.

### The clustering coefficient of the PPI network

**Figure 2:**
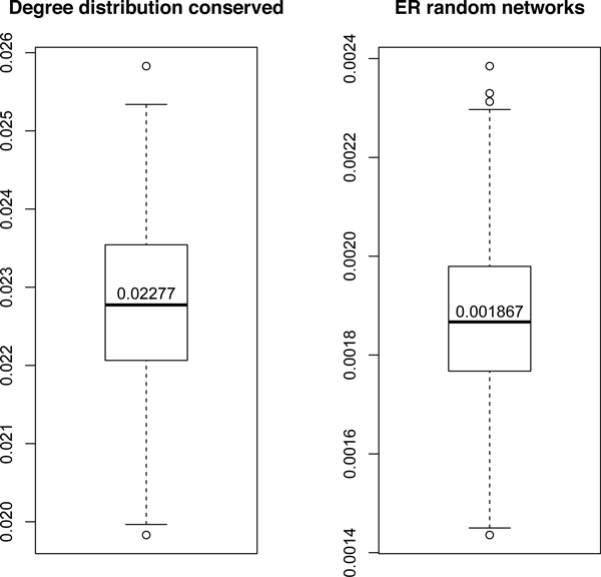
**S.cerevisiae's PPI network shows a relatively larger clustering coefficient**. To make the observed clustering coefficient of the PPI network (0.0970) comparable, two network generation procedures are employed as null models. The clustering coefficients of the null models are shown as boxplots.

The clustering coefficient of the PPI network we used is 0.0970. To make it comparable, two network generation models are employed as null models: the scale-free model [14] and the ER random graph model [25]. 'Scale-free' networks exhibit power-law degree distributions. The ER random graph model *G_n, m_*is a collection of graphs with *n *nodes and *m *edges $\begin{array}{c} \left(m\leq \frac{n\left(n-1\right)}{2}\right) \end{array}$ exactly, and all possible edges in the graphs are distributed uniformly, which is equivalent to connecting the nodes with identical probability $\begin{array}{c} \left(\frac{2m}{n\left(n-1\right)}\right) \end{array}$. Particularly, we generate networks under two null models and estimate the distribution of their clustering coefficient separately. For the 'scale-free' model, the degree distribution of the original network is kept while rewiring the edges. For the ER random graph model, only the number of edges is conserved, and edges are selected randomly. For each model, 500 sample networks are generated, and the distribution of their clustering coefficient is shown as boxplots (Figure 2). The median clustering coefficients are 0.02277 and 0.001867 respectively for the scale-free model and the ER random graph model, from which we can assert that the clustering coefficient of the observed PPI network is significantly large. This validates the start point of our consideration.

### Predicted DDIs are differently enriched in the golden data set

We first evaluate the performance difference between the modified model and the original one through counting the number of domain pairs confirmed by the golden data set. The linear programming problem after relaxation has 30394 variables and 20709 constraints, but there are only 756 variables (DDIs) in the golden data set, due to the difficulties in detecting DDIs experimentally. So we face a problem of lacking 'positives', and thus the rate of false positives may be excessive. But considering that our main purpose here is to investigate the role of the weights, we still expect to see a difference.

Specifically, 'sensitivity' and 'fold change' defined below are used to evaluate the performances of the models.

(15) $$\begin{array}{c} \text{sensitivity}=\frac{True\;Positives}{True\;Positives+False\;Negative} \end{array}$$

(16) $$=\frac{True\;Positives}{756}$$

(17) $$\begin{array}{c} \text{Fold}\;\text{Change}=\frac{True\;Positives}{Total\;Predictions\times \frac{756}{30394}} \end{array}$$

**Figure 3:**
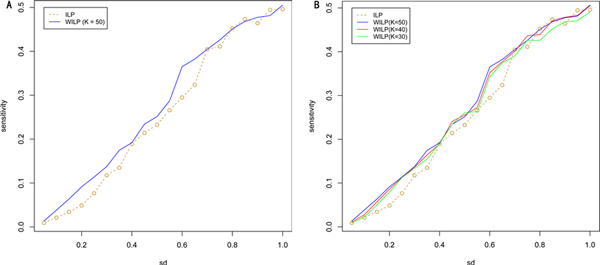
**WILP outperforms ILP in terms of the number of the predicted DDIs confirmed by the golden data set**. (A) Sensitivities of WILP and ILP are compared as *sd *varies from 0.05 to 1. In WILP, *K *is set to 50. (B) Performances of WILP are shown in different *K *settings. There is a broad interval in which WILP outperforms ILP robustly.

The results of WILP model and the ILP model are shown in Figure 3A and Table 1. When the parameter *sd *varies from 0.8 to 1, there's no significant difference in 'sensitivity', but when *sd *varies from 0.05 to 0.7, it can be clearly seen that WILP outperforms ILP, which matches our expectation. For why there is no clear positive signal when *sd *falls in [0.8, 1], we give two possible reasons from a computational point of view. First, as mentioned above, a large proportion of false positives in PPIs may hinder the performance of computational models. Here, when *sd *decreases, the model removes a prescribed proportion of constraints to achieve a most parsimonious subset of PPIs. This process may clean the original observed PPIs because we have proved that the organization of PPIs and protein domain compositions follows a parsimonious way. Second, lacking 'positives' leads to an under-estimation in 'True Positives' (TP). These two reasons can also explain that why the improvement we obtain is slightly weak even when *sd *falls in [0.05,0.75].

**Table 1:** Performance comparison between WILP and ILP

sd	Total Predictions	True Positives	Sensitivity(%)	Fold Change
1	12663 (12663)	382 (375)	50.53 (49.60)	1.21 (1.19)
0.9	10592 (10592)	361 (351)	47.75 (46.43)	1.37 (1.33)
0.8	8521 (8521)	341 (342)	45.11 (45.24)	1.61 (1.61)
0.7	6450 (7102)	306 (306)	40.48 (40.48)	1.91 (1.73)
0.6	4379 (5162)	276 (223)	36.51 (29.50)	2.53 (1.74)
0.5	2648 (3091)	190 (176)	25.13 (23.28)	2.88 (2.29)
0.4	1613 (1620)	145 (143)	19.18 (18.92)	3.61 (3.55)
0.3	875 (779)	104 (89)	13.76 (11.77)	4.78 (4.59)
0.2	430 (279)	69 (37)	9.13 (4.89)	6.45 (5.33)
0.1	131 (63)	29 (16)	3.84 (2.12)	8.90 (10.21)

Comparison of WILP and ILP in terms of the number of the predicted DDIs confirmed by the golden data set. Predicted DDIs verified according to the golden data set are denoted as true positives. 'Sensitivity' and 'Fold Change' are defined in the main text. Numbers marked in red means that WILP outperforms ILP

There is a parameter *K *in the WILP model, which is actually a threshold defining 'poor nodes' and controls the size of *I*_1_. According to the preceding reasoning, a larger *K *results in a smaller *I*_1 _and the extracted prior information is more precise but less. In the numerical experiments, a broad range of *K *are used and the performance is quite robust (Figure 3B).

### Statistical significance of the weights

**Figure 4:**
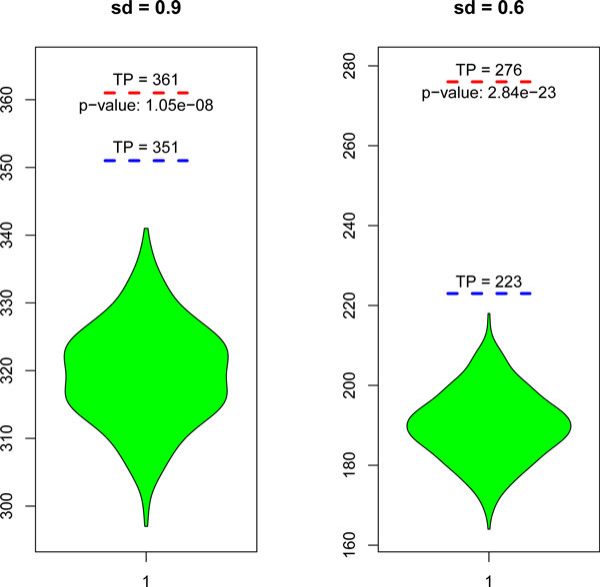
**Statistical significance of the weights**. Random weights are given to WILP and the distributions of 'TP' are shown as 'violin plots'.

The performance difference between WILP and ILP has been shown above. In this section, we confirm that the observed accuracy improvement is not obtained by chance. That is to say, the weights derived from network properties are indeed meaningful. Particularly, random weights are given to WILP (the null model) and the distribution of TP is estimated and compared with real values (Table 1). Specifically, the random weights are generated from a uniform distribution between 0 and 0.5 and the number of weighted domain pairs is the same as the true model. TP is selected as the test statistic because we find that 'Total Predictions' and the weights added are almost independent. The distribution of TP is shown as 'violin plots' (Figure 4), *p*-values are computed using the Gaussian distribution (500 runs for each *sd *setting). There is a significant performance difference between true weights and randomly generated weights (In both cases, *p*-values are smaller than 1.00*e*-5), so we can reasonably assert that the accuracy improvement observed in WILP is a consequence of adding meaningful weights to domain pairs.

### Functional similarity analysis of predicted DDIs

**Figure 5:**
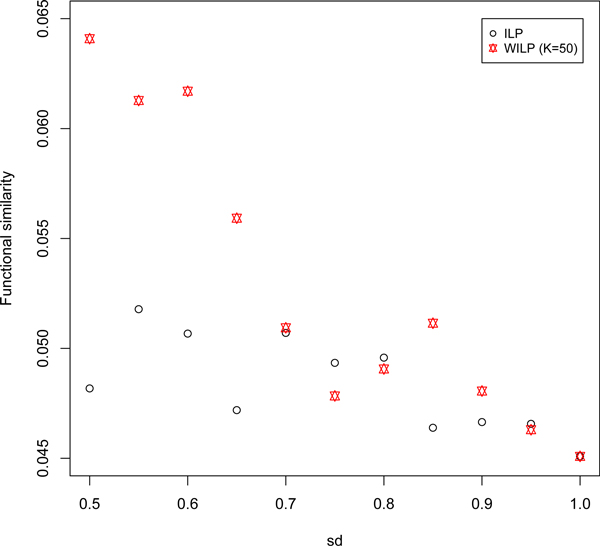
**Similarity analysis of the predicted DDIs**. Comparison of functional similarities of the predicted DDIs obtained by ILP and WILP (*sd *varies from 0.5 to 1).

WILP outperforms ILP in terms of the number of the predicted DDIs confirmed by the golden data set. In this section, these two models are compared in a functional view. In gene expression analysis, co-expression genes are deemed to be functionally similar for they may be involved in a same biological process. It is natural to hypothesize that physical interacting domains have similar biological functions. This impels us to compare WILP and ILP by examining the functional similarity of predicted DDIs. GO terms have been mapped to Pfam entries [26] and domain-domain functional similarity measure is based on similarities of corresponding GO terms. Particularly, GOSim [27] is used to compute similarities between GO terms and for a pair of domains, their similarity is defined as the maximum similarity of involved GO terms. For a set of predicted DDIs, the similarity profile is the average. Because not all domains have mapped GO terms, DDIs which include domains without annotation are dropped. DDIs predicted from WILP show higher functional similarities in general than those predicted by ILP as *sd *varies from 0.5 to 1 (Figure 5). This further validates the biological meanings of the weights extracted from the general properties of the PPI complex network conformation.

## Conclusions

Knowledge about domain-domain recognition patterns provide insights of the organization of PPIs and protein function. While DDIs are difficult to be determined experimentally, many computational approaches have been proposed aiming at discovering the patterns from DDIs, among which parsimony-based models show their advantages in easy implementation and power in detecting specific DDIs. We notice that existing methods only make use of PPIs in a local way. As PPI networks are an important case of complex networks and exhibit global properties such as 'small-world', 'scale-free' and relatively larger clustering coefficient, in this paper, we try to integrate the clustering coefficient feature as prior known knowledge into the computational model.

Results show that WILP outperforms ILP to some extent, which confirms us that those properties are biologically meaningful. This may shed light on a new perspective in studying DDI and PPI networks. Currently, studies of complex networks mainly focus on those common features but few work has been done to investigate what is behind them. We point out that those features can be connected with a specific problem in computational biology. Then we can study the role of the features in a context-depended way, where plenty of tools have been developed.

## Competing interests

The authors declare that they have no competing interests.

## Authors' contributions

XSZ and RSW designed the study. CC, JFZ and QH implemented the method, performed the experiments and analyzed the data. All authors contributed to discussions on the method. CC and XSZ wrote the manuscript. All authors revised the manuscript and approved the final version.
